# Transcatheter atrial septal defect closure in an infant (body weight 6.4 kg) using the GORE CARDIOFORM septal occluder (GCSO)

**DOI:** 10.1186/s40348-017-0077-7

**Published:** 2017-11-03

**Authors:** Roman Scheidmann, Thomas Paul, Matthias Sigler

**Affiliations:** Department of Pediatric Cardiology and Intensive Care Medicine, University Medical Centre, Georg-August-University, Robert-Koch-Str. 40, D-37075 Göttingen, Germany

**Keywords:** Atrial septal defect of secundum type (ASD II), GORE CARDIOFORM septal occluder (GCSO), Transcatheter ASD closure, Interventional therapy, Endovascular procedure

## Abstract

**Introduction:**

Transcatheter closure has become the treatment of choice for secundum atrial septal defects (ASD II), but particularly in small children, there is concern regarding procedure-related complications.

**Case description:**

We report on a 10-month-old infant, body weight of 6.4 kg, with a large ASD who was referred for failure to thrive and dyspnea on exertion. Echocardiography showed two neighboring ASDs centrally located within an atrial septum with a length of 27 mm resulting in significant left-to-right shunting. During cardiac catheterization, hemodynamic significance of the defect as well as normal pulmonary vascular resistance was demonstrated. Balloon sizing of the central ASD showed a stretched defect diameter of 12 × 11 mm. A 20-mm GORE CARDIOFORM septal occluder (GCSO; Goremedical, W. L. Gore & Associates, Inc., Newark, DE, USA) was implanted without any complications. Initial trivial residual shunting resolved during 4 months of follow-up. Right ventricular dimensions declined significantly, and the boy gained body weight properly.

**Discussion, evaluation and conclusion:**

As demonstrated in our report, even large ASDs can be closed safely by catheter intervention in small infants. Selection of implant device and optimal sizing is of paramount importance. The size of the delivery sheath (11 French in our patient) is a potential limitation for the GCSO in smaller infants.

## Background

Transcatheter closure has become the treatment of choice for secundum atrial septal defects. A wide range of occlusion devices is available, but concern has been raised about procedure-related adverse events, especially cardiac erosion and vascular damage, with an increased risk in small patients [1].

## Case description

We report the case of an infant with Down’s syndrome who underwent transcatheter ASD closure using the GORE CARDIOFORM septal occluder (GCSO) with 6.4 kg body weight. The boy had been referred due to failure to thrive and dyspnea on exertion.

Echocardiography showed two neighboring ASDs centrally located within the atrial septum with a length of 27 mm resulting in significant left-to-right shunting, significant right heart enlargement, and functional pulmonary stenosis.

**Fig. 1:**
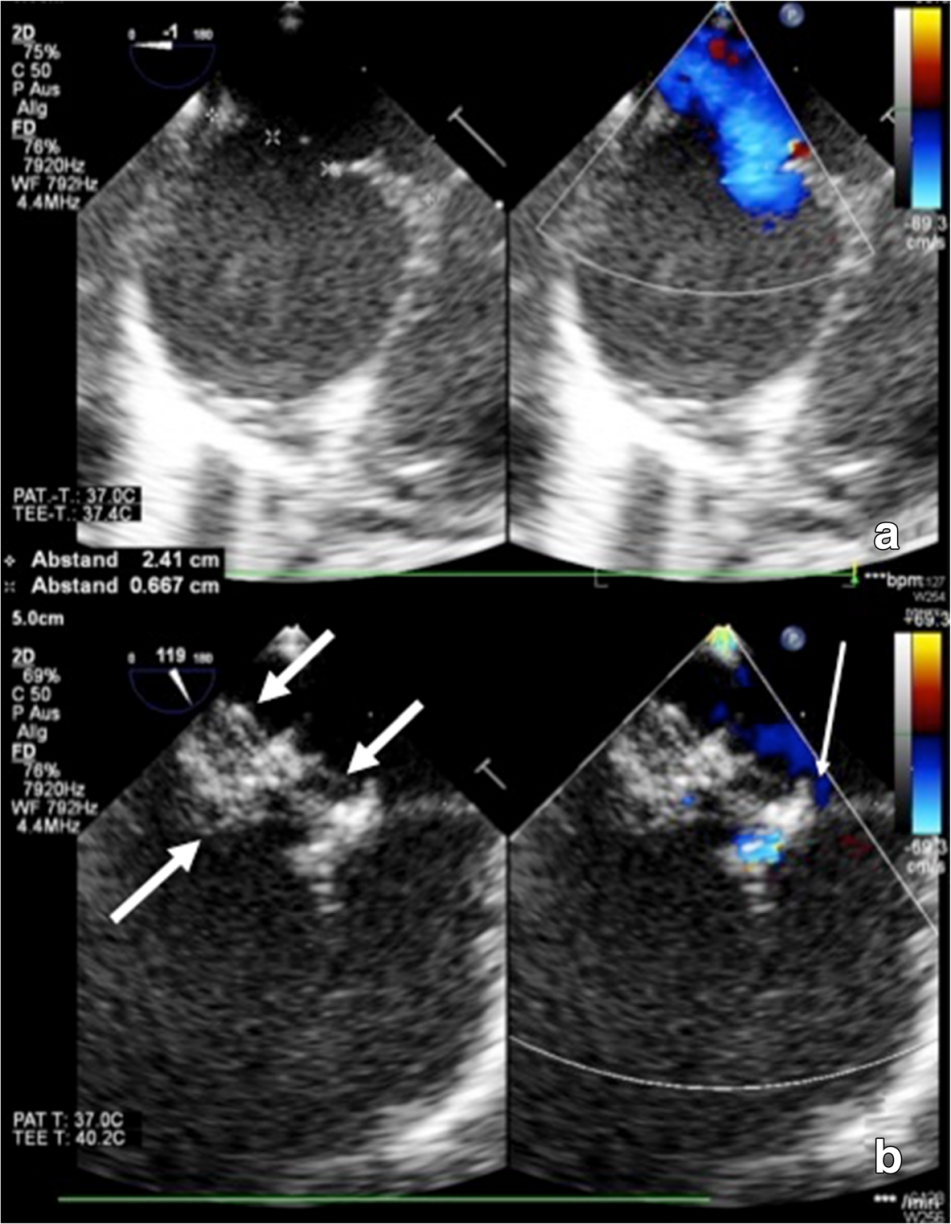
Transoesophageal echocardiography during interventional ASD closure. Echocardiographic measurement of the larger ASD prior to interventional closure (**a**). Echocardiographic image after successful implantation of a GCSO (bold arrows) into the larger ASD. Incomplete coverage of the smaller ASD resulted in trivial residual shunting (slim arrow) (**b**)

**Fig. 2:**
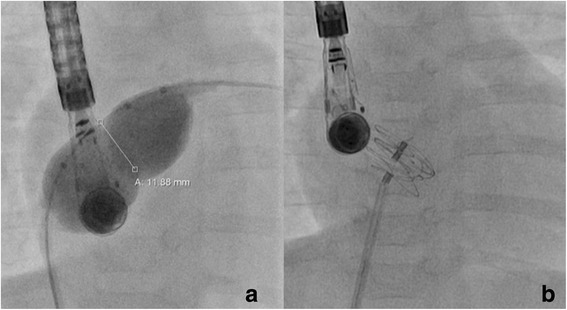
Balloon sizing of ASD and GCSO implantation. Anterior-posterior fluoroscopic view with inflated sizing ballon (“ballon sizing”) of the larger of the two ASDs while simultaneous transoesophageal echocardiography showed no residual shunting through this defect (**a**). Completely developed device in projection to the atrial septum, still attached to the delivery catheter (**b**)

Due to persistent failure to thrive at 10 months of age, the baby underwent cardiac catheterization under general anesthesia. Catheter intervention was guided by transoesophageal echocardiography and fluoroscopy (Fig. 1). Balloon sizing of the central ASD demonstrated a stretched defect diameter of 12 × 11 mm (Fig. 2). The smaller, craniodorsally located defect measured 4 × 2 mm. Hemodynamic evaluation confirmed significant left-to-right shunting (pulmonary flow/systemic flow (Qp/Qs) = 2.5:1).

Initially, it was decided to implant a 25-mm GCSO in order to cover both ASDs. The device was loaded into the sheath after careful flushing with normal saline. Before introducing, a guide wire had been placed into the upper left pulmonary vein through an 11 French introducer sheath (Terumo Corp., Tokyo, Japan). The sheath with the loaded device was then forwarded using the monorail technique. After unfolding of the left atrial disc in the left atrium, the cranial edge of the disc was repetitively slipping through the defect into the right atrium due to the system’s intrinsic stiffness and an angle of 45° between the atrial septum plane and the application sheath. Therefore, it was decided to switch to a smaller 20-mm device albeit its potential risk of residual shunting. Device implantation was performed into the larger ASD without any complications but trivial residual shunting (Qp/Qs = 1.2:1) through the smaller ASD (residual defect size 2 × 1 mm) due to incomplete device coverage. Fluoroscopy time was 16 min.

The baby received periprocedural antibiotic therapy with cefazolin (100 mg/kg). For avoiding clot formation, unfractionated heparin (200 IE/kg) was given for the first 48 h followed by acetyl salicylic acid for 6 months.

No procedure-related complications were documented by follow-up routine diagnostic examinations (electrocardiogram, transthoracic echocardiography and duplex-sonography of femoral veins), particularly no electrocardiographic alterations, cardiac erosion, pericardial effusion, or vascular damage at the access site*.* Correct position of the device and initial residual shunting was documented by routine transthoracic echocardiography. Within the following weeks, right ventricular dimensions diminished significantly. The boy thrived properly. Four months after interventional closure, no residual shunt was detectable by transthoracic echocardiography.

## Discussion and evaluation

As demonstrated in our report, large ASDs can be closed safely by catheter intervention even in small infants. Bishnoi et al. analyzed technical aspects and complications related to interventional ASD closure in 68 infants < 8 kg from 10 different hospitals in the USA. Data suggest that interventional ASD closure was safe and effective, resulting in significant clinical improvement [2]. Data from Wyss et al. and Abu-Tair et al. support these findings reporting 14 and 28 infants with a body weight < 10 kg respectively who underwent interventional ASD closure without any major complications [3, 4].

Selection of type and optimal size of the device may sometimes be challenging. In the face of the small size of the baby and potential mechanical tissue trauma, the use of the GCSO was favored due to its more soft and flexible design compared to metal–mesh-based occlusion devices such as the Amplatzer Septal Occluder (Abbott, St. Paul, MN). As demonstrated in our report, it might be necessary to switch to a different device size in order to safely position the implant in the defect.

Furthermore, over- and undersizing should be avoided whenever possible since inappropriate size of the occlusion device was identified as a main risk factor for cardiac erosion and perforation respectively [5].

Even if complete closure of the defect cannot be achieved initially, the clinical status is often improved by reducing shunt volume to a hemodynamically insignificant level. Small ASDs, as in our patient, have a high rate of spontaneous closure [6].

## Conclusion

The size of the introducer sheath (11 French in our patient) is probably the main limiting factor for the GCSO in infants. This problem can be addressed by a new delivery technique where the delivery sheath serves as the introducer so that vascular access size requirement can be reduced by 25% [7]. In our case, we decided to use the GCSO because of its more soft and flexible design compared to the Amplatzer septal occluder, as mentioned above. We wanted to primarily reduce any risk of potential cardiac trauma by the device. This aspect was given priority over a possible vascular damage by a large introducer system.

On the other hand, the definite risks and inherent complications of open-heart surgery under cardio-pulmonary bypass can be avoided by using an interventional approach in the therapy of a hemodynamically significant ASD [8].

## Abbreviations

ASD: Atrial septal defect
GCSO: GORE CARDIOFORM septal occluder
Qp/Qs: Qp = Pulmonary flow. Qs = Systemic flow. The ration Qp/Qs describes the magnitude of a cardiovascular shunt
